# Three-dimensional cell culture for the study of nasal polyps

**DOI:** 10.1016/j.bjorl.2021.11.001

**Published:** 2021-11-26

**Authors:** Carolina Nunes França, André Luis Lacerda Bachi, Eduardo Macoto Kosugi, Rogério Pezato, Gláucia Maria Machado Santelli, Jônatas Bussador do Amaral

**Affiliations:** a Universidade de Santo Amaro (UNISA), Programa de Pós-Graduação em Ciências da Saúde, São Paulo, SP, Brazil; b Universidade Federal de São Paulo, Departamento de Otorrinolaringologia e Cirurgia de Cabeça e Pescoço, Laboratório de Pesquisa ORL, São Paulo, SP, Brazil; c Universidade de São Paulo, Departamento de Otorrinolaringologia e Oftalmologia, São Paulo, SP, Brazil; d Universidade de São Paulo, Instituto de Ciências Biomédicas, Departamento de Biologia Celular e do Desenvolvimento, São Paulo, SP, Brazil

**Keywords:** 3D cell culture, Spheroids, Chronic Rhinosinusitis with Nasal Polyps, Confocal microscopy, Morphology

## Abstract

**Objectives:**

Three-dimensional (3D) cell cultures have many applications such as stem cell biology research, new drug discovery, cancer, and Chronic Rhinosinusitis with Nasal Polyps (CRSwNP). This disease is characterized by a significant impact on quality of life and productivity. The diversity of factors that act in the progression of CRSwNP point to the creation of a cell culture model that allows the integration of different cell types with extracellular matrix. This work aimed to create a cell culture model in 3 dimensions (spheroids) for the study of Nasal Polyposis.

**Methods:**

Nasal polyp tissue from patients diagnosed with CRSwNP was mechanically dissociated using tweezers and a scalpel and the solution containing cells and small aggregates of nasal polyps was transferred to a Petri dish containing 5 mL of culture medium at the concentration of 10^6^ cells/mL.

**Results:**

The spheroids were cultivated for 20 days, fixed and analyzed using confocal microscopy. In a 3D culture environment, the spheroids were formed both by clustering cells and from small tissue fragments. In the cultures analyzed, the ciliary beat was present from the dissociation of the cells up to 20 days in culture.

**Conclusion:**

Our findings also point to these characteristics showing the environment generated in our study, the cells remained differentiated for a longer time and with ciliary beating. Thus, this work shows that nasal polyp-derived cells can be maintained in a 3D environment, enabling better strategies for understanding CRSwNP in situations similar to those found *in vivo*.

**Level of evidence:**

Laboratory studies.

## Highlights

• Cells in a 3D dimensional environment are more similar to *in vivo* than a monolayer.
• In 3D environment the cells remained differentiated for a longer time.
• Nasal polyp spheroids can be used to understand CRSwNP.

## Introduction

Chronic Rhinosinusitis with Nasal Polyps (CRSwNP) is a prevalent condition characterized by a significant impact on quality of life and productivity. These nasal polyps are benign inflammatory masses, arising from the mucosa of the nose and paranasal sinuses^1^ with frequent recurrence after both medical and surgical treatment.^2^, ^3^, ^4^ Two main pillars support nasal polyp development: a chronically inflamed environment associated with a mechanical dysfunction.^5^ A major obstacle to the study of CRSwNP is the lack of experimental models that mimic nasal polyposis. Animal models fail to represent the different endotypes found in CRSwNP, and the epithelial monolayer culture is limited to a single cell type. Due to the difficulties to study nasal polyps, it is proposed to use bronchial mucosa as a model to understand the mechanism involved in CRSwNP development.^6^

Monolayer cell cultures are important in scientific research. The reproducibility of the experiments and their large-scale use by different laboratories made this technique fundamental in the production of vaccines,^7^ in the compression of cell biology,^8^ and studies with cancer.^9^, ^10^ Despite this undeniable importance, the two-dimensional (2D) culture model has limitations that distance it from *in vivo* models^11^ mainly due to less cell-cell and matrix cell interaction,^12^ limited cell heterogeneity, and formation of the microenvironments.^13^, ^14^

As it allows an increase in the complexity of interactions, the three-dimensional (3D) cell cultures are an important tool for *in vitro* models. Cells can be grouped in several layers when subjected to this environment, which exposes them differently both between the cells and with the matrix components. As a consequence, we can observe in 3D cell cultures gaseous perfusion gradients,^15^ unequal distribution of metabolites,^16^ differentiated exposure to drugs,^17^ differences in gene expression, and consequent differentiation of cell populations. In addition, the incorporation of different cell types in this 3D system generates physiologically relevant results that emulate tissue healthy or in different pathological conditions.^18^

There are different ways to obtain 3D cell cultures, within these techniques, spheroid culture is characterized by an aggregation of cells originating from a single cell or a mixture of cells. These cellular aggregates are generated from primary cells or established cell lines of normal and tumor specimens with or without an extracellular matrix.^19^ Three-dimensional cell cultures have many applications such as stem cell biology research, new drug discovery, cancer, and other disease studies^11^ such as, for example, chronic rhinosinusitis.^20^

The diversity of factors that act in the progression of this disease point to the creation of a cell culture model that allows the integration of different cell types with extracellular matrix components for a better understanding of this pathology. Therefore this work aimed to create a cell culture model in 3 dimensions (spheroids) for the study of CRSwNP.

## Methods

This study was approved by the Ethical Committee of Federal University of Sao Paulo (number 0305/2019). Informed consent was obtained from each subject, under the Helsinki Declaration.

### Patients and clinical diagnosis

A total of 8 individuals with CRSwNP were included in the study (Table 1). The diagnosis of the disease was performed according to the European position paper on Rhinosinusitis and Nasal Polyps.^3^

**Table 1:** Demographic data and clinical characteristics of the study population.

Patients with CRSwNP (n)	8
Male, n (%)	5 (62.5)
Age, years (mean ± SD)	56.5 ± 9.8
Asthma, n (%)	4 (50)
Aspirin intolerance, n (%)	2 (25)
Current smoker, n (%)	1 (12.5)

SD, standard deviation; CRSwNP, Chronic Rhinosinusitis with Nasal Polyps.

### Isolation of nasal polyps

Nasal polyp tissue from patients diagnosed with CRSwNP was collected during scheduled Endoscopic Sinus Surgery (ESS) at the Hospital in Federal University of Sao Paulo. Tissue specimens were aseptically collected and transported to the ENT research Lab in tubes containing 5 mL of Dulbecco’s modified Eagle’s medium (DMEM) in a refrigerated box at 8°–12 °C. In a sterile environment, the Nasal Polyp fragments were washed three times with Phosphate Buffered Saline (PBS).

### Spheroids of nasal polyp cells

The technique used for spheroid formation was cell culture with liquid overlay, which consisted of preventing adhesion on the surface of the plate.^12^ Tissue fragments were cut into fractions containing 2 mm^2^ and were transferred to a Petri dish where 50 µL of BEBM (Bronchial Epithelial Cell Basal Medium) with Single Quots kit (Clonetics, Lonza Group Ltd., Switzerland).^21^ A 1:1 solution of this medium + DMEM (Dulbecco’s Modified Eagle’s Medium — Sigma) was used in all experiments (adapted from).^21^ Each fragment was mechanically dissociated using tweezers and a scalpel. The solution containing cells and small aggregates of nasal polyps was transferred to a Petri dish containing 5 mL of the same culture medium at the concentration of 10^6^cells/mL. Plates containing spheroids were kept at 37 °C with 5% CO_2_. Every 3 days, the dish contents were poured into conical centrifugation tubes (15 mL). By gravity, spheroids are concentrated at the bottom of the tubes. This procedure enabled the spheroids to separate from the culture medium, and the spheroids were then washed and plated in a new culture medium. The spheroids were cultivated for 20 days.^12^

### Monolayer cell culture

Tissue fragments were cut into 0.3–0.5 mm and placed on the surface of cell culture flasks. After the adhesion of the fragments, BEBM with Single Quots kit + DMEM (1:1) were added. Culture flasks were incubated at 37 °C in a humidified atmosphere of 5% CO_2_.^22^

### Light microscopy

Digital images were obtained at all development stages of the spheroids by both phase contrast and bright field microscopy using an inverted microscope (Nikon TS100) at 400× magnification with a digital camera. The area of spheroids (1 day) was measuring with ImageJ software (version 1.52) (National Institutes of Health, Bethesda, MD, USA).

#### Confocal laser scanning microscopy

Spheroids were fixed in 3.7% formaldehyde in PBS for 30 min. After washing with PBS, the cells were treated with 0.5% Triton-X 100 for 30 min (under agitation). After another wash, the cells were blocked using 3% albumin in PBS. After 1 h, cells were once again washed with PBS and submitted to immunofluorescence. The primary antibodies, anti-α and β tubulin (Sigma) were used in 1:250 concentration and added 30 µL in 1.5 mL tubes for 12 h under agitation. After another wash with PBS, the aforementioned procedure was repeated for application of secondary antibodies, Anti-IgG mouse or rabbit–Cy5, with 6 h under agitation. Actin microfilaments were stained with 7.5 mM FITC-phalloidin (Sigma) for 4 h and the cells were then washed with PBS. After treatment with RNase (10 mg/mL) for 1 h, nucleus staining was performed using propidium iodide at a concentration of 10 µg/mL (Sigma). The coverslips containing Nasal Polyps Spheroids were mounted on histological glass slides with an anti-fading solution (Vectashield). The slides were sealed with nail polish and kept in a dark box at −20 °C. Cell analysis was done using confocal laser scanning microscopy (Zeiss LSM 510), and fluorescent images were acquired using the argon green (458, 488, and 514 nm), Helium–Neon1 (543 nm), and Helium–Neon2 (633 nm) lasers. Optical slices were obtained at adequate intervals on the Z-axis (between 0.5 and 1 mm). Different modules of the LSM 510 3D software (Carl Zeiss) were used in the confocal analysis, including slice projections, orthogonal projections, and animations. Some reconstructions and animations were made using the IMARIS 7.1 software (Bitplane).^12^, ^13^

## Results

**Figure 1:**
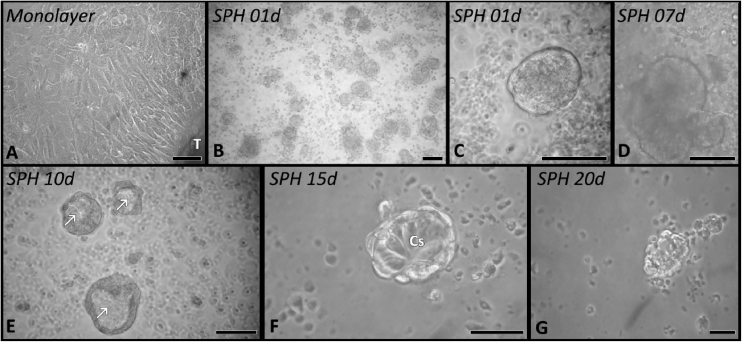
Phase contrast images of nasal polyp-derived cells. (A) Cells spreading from a tissue fragment (T) forming a monolayer of cells. (B–G) 3D cell culture (spheroids), the sequence of images shows the dynamics of spheroid formation from day 1 (B) keeping viable until the 20th day of culture (G). In the spheroids from the 10th day (E) it was observed in some cell aggregates the formation of a cavity in the medullary region (arrows). CS, columnar cells; Barr 50 µm.

Nasal polyp-derived cells were maintained in cell cultures in either monolayer (Fig. 1A) or 3D culture (Fig. 1B–G).

In a monolayer, the cells gradually migrated from the small tissue fragments and spread through the culture bottle, presenting an epithelial morphology (Fig. 1A). The maintenance of some epithelial functionalities, such as the presence of ciliary beats (Supplementary material), was observed. However, around the 7th day of culture, both cell spreading and ciliary beats are less frequent.

In a 3D culture environment (Fig. 1B–G) the spheroids were formed both by clustering cells and small tissue fragments. In 24 h of culture, it was possible to observe the characteristic spherical arrangement of these structures (Fig. 1B–C), having an average area of 80 µm^2^ (SD ± 43). In the cultures analyzed, the ciliary beat was present from the dissociation of the cells up to 20 days in culture (Supplementary material).

The distribution of cells inside the spheroid was heterogeneously organized over time. Initially, all spheroids had a spherical arrangement and cells with strong interaction with each other (Fig. 1B–D). Around the 10th day, this arrangement was replaced in some spheroids by a cavity-like structure (Fig. 1E), with the cells now arranged in the cortical region. The morphology of the cells inside the spheroid varied from a cuboid, columnar and flat cell arrangement in the cortical region (Fig. 1F). Between periods of 15–20 days of culture, the 3D cell culture model begins to lose viability as it presents a gradual decrease in the number of cells associated with a gradual dissociation of their three-dimensional arrangement (Fig. 1G).

**Figure 2:**
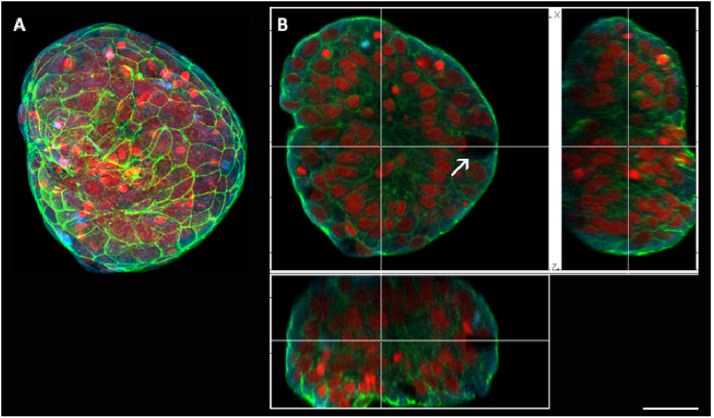
Scanning confocal microscopy of spheroids of nasal polyp-derived cells with 7 days of culture. (A) 3D reconstruction of a spheroid with 7 days of culture. (B) Orthogonal sections of the previous image, where it is possible to observe the interaction of cells inside the spheroid. Goblet cell (arrow). In red it is possible to visualize the nuclei of the cells (propidium iodide), in green the actin filaments (FITC-phalloidin) and the microtubules-alpha and beta tubulin in blue (Cy5). Barr 30 µm.

**Figure 3:**
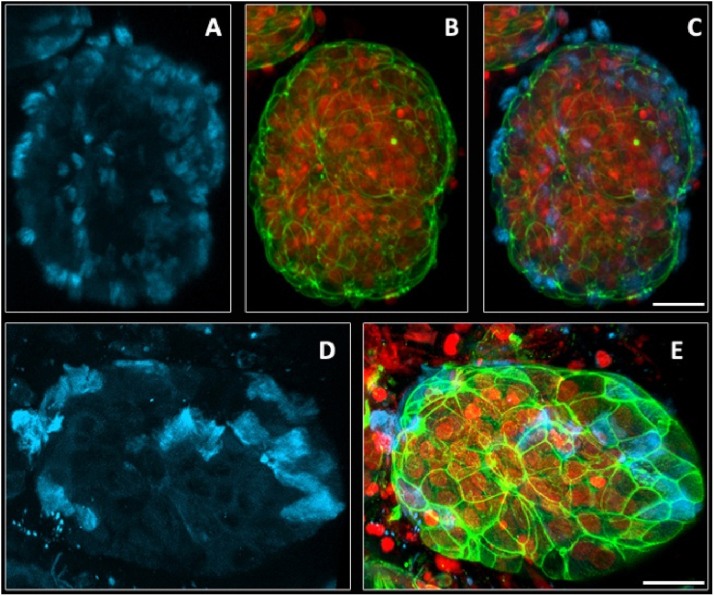
Scanning confocal microscopy of spheroids of nasal polyp-derived cells with 7 days of culture. (A–E) 3D reconstruction of a spheroid with 7 days of culture. In images (A and D) it is possible to observe the distribution of microtubules inside the cells. Fig. B it is possible to observe the distribution of F actin in the cells inside the spheroid. In Figs. C and E the images are merged (actin-F and Tubulin). In red it is possible to visualize the nuclei of the cells (propidium iodide), in green the actin filaments (FITC-phalloidin) and the microtubules-alpha and beta tubulin in blue (Cy5). Barr 30 µm.

Spheroids after 7 days of culture were also analyzed using confocal microscopy (Figure 2, Figure 3). In orthogonal sections, it was possible to observe the presence of cells with an arrangement very similar to goblet cells, with a basal nucleus and absence of fluorescence in the cytoplasm (Fig. 2B). Cellular interaction inside the spheroid was also confirmed by optical sections, with no cavities being found inside the spheroid (Fig. 2B).

The F-actin filaments showed a homogeneous distribution in the cytoplasm (Figs. 2A, 3B and E) of the cells inside the spheroids. There was a higher concentration of these filaments in the contact regions between cells, increasing the fluorescence intensity (Figs. 2A and 3E).

Immunofluorescence for alpha and beta-tubulin also showed heterogeneous microtubules organization in the cell cytoplasm (Fig. 3A and D). In 3D confocal image reconstructions, it was possible to visualize the distribution of cilia in the apical region of the Ciliary cells (Fig. 3A and D), confirming the presence of these structures seen in the movies (Supplementary material). The presence of Ciliary cells occurred in all experiments (Supplementary material); however, their presence is not mandatory in all spheroids (compare Fig. 2A with Fig. 3D and see the Supplementary material).

## Discussion

The lack of experimental models that represent the unique chronic inflamed environment associated with an altered composition of extracellular matrix found in nasal polyps makes it necessary to develop new experimental models that better resemble the polypoid tissue found in the nose. Our results show that nasal polyp-derived cells can be used in a 3D cell culture. This model for the formation of spheroids has been used by our group in the context of tumor cell biology.^12^, ^13^, ^14^ Growing in suspension, these cells avoid direct physical contact with the plastic dish and remained viable spheroids for 20 days. The use of this technique is simple, economical, highly reproducible, and adaptable.^23^ becomes indispensable for *in vitro* modeling of fundamental developmental processes as well as human diseases. Increasing complexity has led to more advanced approaches in personalized medicine and drug testing.^18^

The maintenance of homeostasis of the nasal and paranasal sinuses is largely provided by the nasal mucosa. The epithelium in this region is made up of basal, goblet, and columnar cells that may or may not have cilia.^24^ However, the inflammatory processes associated with CRSwNP can lead to damage to the epithelium and abnormal remodeling can also occur.^25^ We reproduced this cellular heterogeneity, with spheroids with different amounts of cilia and cell types. Columnar cells also organized over culture time, assuming a more polarized arrangement in some spheroids. This formation was also found in spheroids originated from nasal epithelium brushing, which also adds a basolateral-apical polarity in the cortical region and with the formation of cystic structures.^26^ Such characteristics point to the possibility of using spheroids derived from nasal polyps as a study model for the control of water flow, electrolytes, and epithelium junctions.

3D airway epithelial cell culture exhibits advantages when compared to 2D cell culture in that it allows repolarization and maintenance of functional apical membrane-associated proteins.^20^, ^27^ Our findings also point to these characteristics showing the environment generated in our study, the cells remained differentiated for a longer time and with ciliary beating. In terms of applicability, we demonstrated a cheap and easy to reproduce model where it is possible to assess the tissue structural elements and the remodeling process response during the stimulus.

## Conclusion

This work shows that nasal polyp-derived cells can be maintained in a 3D environment, enabling better strategies for understanding CRSwNP in situations similar to those found *in vivo*.

## Conflicts of interest

The authors declare no conflicts of interest.

## Acknowledgements

This work was supported by Conselho Nacional de Desenvolvimento Cientifico e Tecnológico (CNPq Process number 407793/2018-6) and Coordenação de Aperfeiçoamento de Pessoal de Nível Superior (CAPES) PROAP. The authors wish to thank José Arruda Neto for biological samples.

## Appendix A Supplementary data

Video S1 Collection of movies of cells derived from nasal polyps in a monolayer environment (2D) and in three-dimensional cell culture (3D).
